# Iliopsoas Abscess Related to Pseudomonas aeruginosa: A Case Report

**DOI:** 10.7759/cureus.72463

**Published:** 2024-10-27

**Authors:** Kohei Ueda, Koji Hayashi, Shin-ichiro Azuma, Maho Hayashi

**Affiliations:** 1 Department of Internal Medicine, Fukui General Hospital, Fukui, JPN; 2 Department of Rehabilitation Medicine, Fukui General Hospital, Fukui, JPN

**Keywords:** bacteremia, gram-negative bacteremia, hematogenous, iliopsoas abscesses, infective discitis, pseudomonas aeroginosa, secondary iliopsoas abscess, vertebral-osteomyelitis

## Abstract

We describe a rare case of an 80-year-old male with an iliopsoas abscess (IPA) associated with *Pseudomonas aeruginosa* (*P. aeruginosa*). The patient had a history of diabetes mellitus and was admitted to our hospital due to aspiration pneumonia, where he was treated with ampicillin/sulbactam (ABPC/SBT). After admission, he experienced a recurrence of aspiration pneumonia, and ABPC/SBT was repeatedly used. The fever resolved by day 30 and antibiotic therapy was completed on day 33. Although the patient remained afebrile thereafter, anorexia persisted. On day 57, the patient experienced chills, fever, lower back pain, and bowel incontinence, leading to the resumption of ABPC/SBT at 6 g/day. Blood tests on day 59 showed elevated lactate dehydrogenase (239 IU/L) and C-reactive protein (15.08 mg/dL), along with decreased red blood cell count, hemoglobin, and albumin. An abdominal CT scan on day 60 indicated a low-density area suggestive of an abscess in the right iliopsoas muscle, and blood cultures from day 57 were positive for *P. aeruginosa*, prompting a switch to meropenem (MEPM) at 3 g/day. On day 61, lumbar MRI indicated hyperintensity at the L2/3 disc and vertebral bodies, suggestive of discitis and vertebral osteomyelitis. The antibiotic regimen was then changed to ciprofloxacin (CPFX) at 800 mg/day on day 62. Despite ongoing treatment, the patient's fever persisted, and percutaneous and surgical drainage were deemed unfeasible due to the abscess's size and location. The patient experienced a recurrence of pneumonia, leading to a switch to cefepime (CFPM) at 2 g/day on day 86, followed by piperacillin/tazobactam (PIPC/TAZ) at 13.5 g/day on day 96. Due to the deterioration of his clinical condition, he was transferred to a chronic care facility for palliative management on day 102 of hospitalization.

Reports of IPA related to *P. aeruginosa* are very limited. In our case, the patient experienced recurrent pneumonia following hospitalization, and *P. aeruginosa *was isolated from the blood, suggesting that the lungs were the portal of entry, potentially leading to IPA as a result of the bloodstream infection. In cases involving the combination of *P. aeruginosa* and IPA, various compromised host factors, along with *P. aeruginosa* itself, may contribute to adverse outcomes. This report may enhance our understanding of the relationship between IPA and *P. aeruginosa* infections. Further accumulation of case reports and studies is necessary to better understand future treatment strategies and prognosis for IPA related to *P. aeruginosa*.

## Introduction

Iliopsoas abscess (IPA) is a rare condition, with a prevalence of 0.4 cases per 100,000 individuals in the United Kingdom [1]. IPA is an infectious disease characterized by a triad of fever, back pain, and gait disturbance; however, it is rare for all symptoms to present simultaneously. Moreover, other symptoms, such as weight loss and fatigue, are often nonspecific [2]. There are regional differences, with over 90% of cases being primary in Asia and Africa, while in the EU, only around 19% of cases are primary [3].

There are two types of IPA: primary, which occurs via hematogenous or lymphatic spread of bacteria, and secondary, which arises from direct spread from an infectious or inflammatory lesion in nearby tissues [4]. *Staphylococcus aureus* (*S. aureus*) is the most commonly identified in 88% of primary IPA, followed by *Streptococci* (5%) and* Escherichia coli* (*E. coli*) (3%) [4]. In secondary IPA, cultures are often polymicrobial, with* E. coli *and *Bacteroides* species being the most frequently isolated [4]. Other organisms include enteric pathogens, *Staphylococcus* species,* Streptococcus* species, *Mycobacterium tuberculosis*, *Enterococcus faecalis*, and *Peptostreptococcus* [4-7]. Notably, it has been reported that the risk of severe illness is higher when blood cultures are positive, and *E. coli* is identified as the causative pathogen [5].

For diagnosis of IPA, a thorough medical history, physical examination, blood tests, blood cultures, and imaging studies, including magnetic resonance imaging (MRI) and computed tomography (CT), are necessary. MRI has been shown to be more sensitive than computed tomography CT in intra-abdominal abscesses, as CT scans may yield false negatives in cases where the abscess does not contain air or is not clearly defined [8]. Nevertheless, CT scan is valuable in planning treatments for IPA, such as drainage [5]. For empirical treatment, broad-spectrum antibiotics targeting common pathogens, including *S. aureus*, are recommended [5]. Once the causative bacteria have been identified, de-escalation to a narrow-spectrum antibiotic is advised [5]. Although drainage can be employed in IPA management, its effectiveness has not been conclusively demonstrated [5]

*Pseudomonas aeruginosa* (*P. aeruginosa*) is a gram-negative bacillus commonly associated with nosocomial infections, including pneumonia, surgical site infections, urinary tract infections, and bloodstream infections [9, 10]. It is a significant pathogen in healthcare settings, contributing to 7.1% to 7.3% of all healthcare-associated infections [9, 10]. It is well-known that *P. aeruginosa* infections tend to have poor prognosis. The 30-day mortality rate for bloodstream infections caused by *P. aeruginosa* is 34%, and the 90-day mortality rate is 45% [11]. Compared to other pathogens including *E. coli*, the mortality rate of *P. aeruginosa* infections is significantly higher after adjusting for background factors [11]. As far as we know, there have been only three reported cases of iliopsoas abscesses related to* P. aeruginosa* [5,6,12]. In this report, we present a rare case of iliopsoas abscess related to *P. aeruginosa*.

## Case presentation

**Figure 1:**
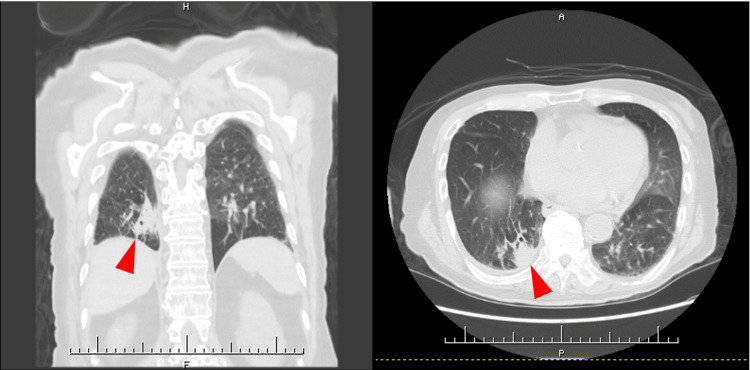
Chest computed tomography (CT) imaging on admission. Chest CT showing infiltration in the lower lobe of the right lung (arrowheads).

The patient is an 80-year-old male who developed chills and difficulty walking due to muscle weakness in the lower extremities one day before admission and presented to our hospital. His medical history includes hypertension, diabetes mellitus, peripheral artery disease of the lower extremities, cerebral infarction, right upper lobectomy by lung cancer, interstitial pneumonia, and aspiration pneumonia, for which he was receiving treatment at a local clinic. Despite these conditions, he maintained independence in his activities of daily living (ADL). Upon arrival at the hospital, his vital signs revealed a temperature of 39.3℃, heart rate of 101 beats/minute, blood pressure of 122/58 mmHg, and SpO₂ of 92% on room air. A cardiovascular examination revealed regular heart sounds with no murmurs, and respiratory sounds were clear with no adventitious sounds. Blood tests revealed an elevated blood glucose level of 181 mg/dL, a hemoglobin A1c of 6.4%, and a C-reactive protein level of 0.27 mg/dL, along with decreased red blood cell count (333×10⁴/μL), hemoglobin (10.3 g/dL), platelet count (13.4×10⁴/μL), and albumin (3.4 g/dL) (Table 1). Chest CT imaging revealed an infiltration in the lower lobe of the right lung (Figure 1). A sputum culture revealed only normal oral flora. Based on these findings, the patient was diagnosed with aspiration pneumonia, and treatment with ampicillin/sulbactam (ABPC/SBT) at 6 g/day was initiated. By day three of hospitalization, the patient became afebrile. On day six, the antibiotic regimen was switched to oral amoxicillin (1500 mg) and clavulanic acid (375 mg), and antibiotics were discontinued on day 11 after his condition had stabilized.

**Table 1:** The results of blood tests on admission.

Inspection items	Result	Reference range
White blood cell count	5800 /μl	(3300-8600)
Red blood cell count	333×104 /μl	(435-555×104)
Hemoglobin	10.3 g/dl	(13.7-16.8)
Blood platelet	13.4×104 /μl	(15.8-34.8)
Total protein	6.8 g/dl	(6.6-8.1)
Albumin	3.4 g/dl	(4.1-5.1)
Alkaline phosphatase	94 U/l	(38-113)
Aspartate aminotransferase	17 U/l	(13-30)
Alanine aminotransferase	12 U/l	(10-30)
Lactate dehydrogenase	153 U/l	(124-222)
Creatine kinase	50 U/l	(59-248)
γ-glutamyltransferase	27 /U/l	(13-64)
Total bilirubin	0.6 mg/dl	(0.4-1.2)
Amylase	121 U/l	(44-132)
Blood urea nitrogen	11.1 mg/dl	(8.0-20.0)
Choline esterase	168 U/l	(240-486)
Creatinine	0.94 mg/dl	(0.65-1.07)
Sodium	138 mmol/l	(138-145)
Potassium	4.7 mmol/l	(3.6-4.8)
Chlorine	108 mmol/l	(101-108)
Glucose	181 mg/dl	(73-109)
Hemoglobin A1c	6.4%	(4.9-6.0)
C-reactive protein	0.27 mg/dl	(0.00-0.14)

**Figure 2:**
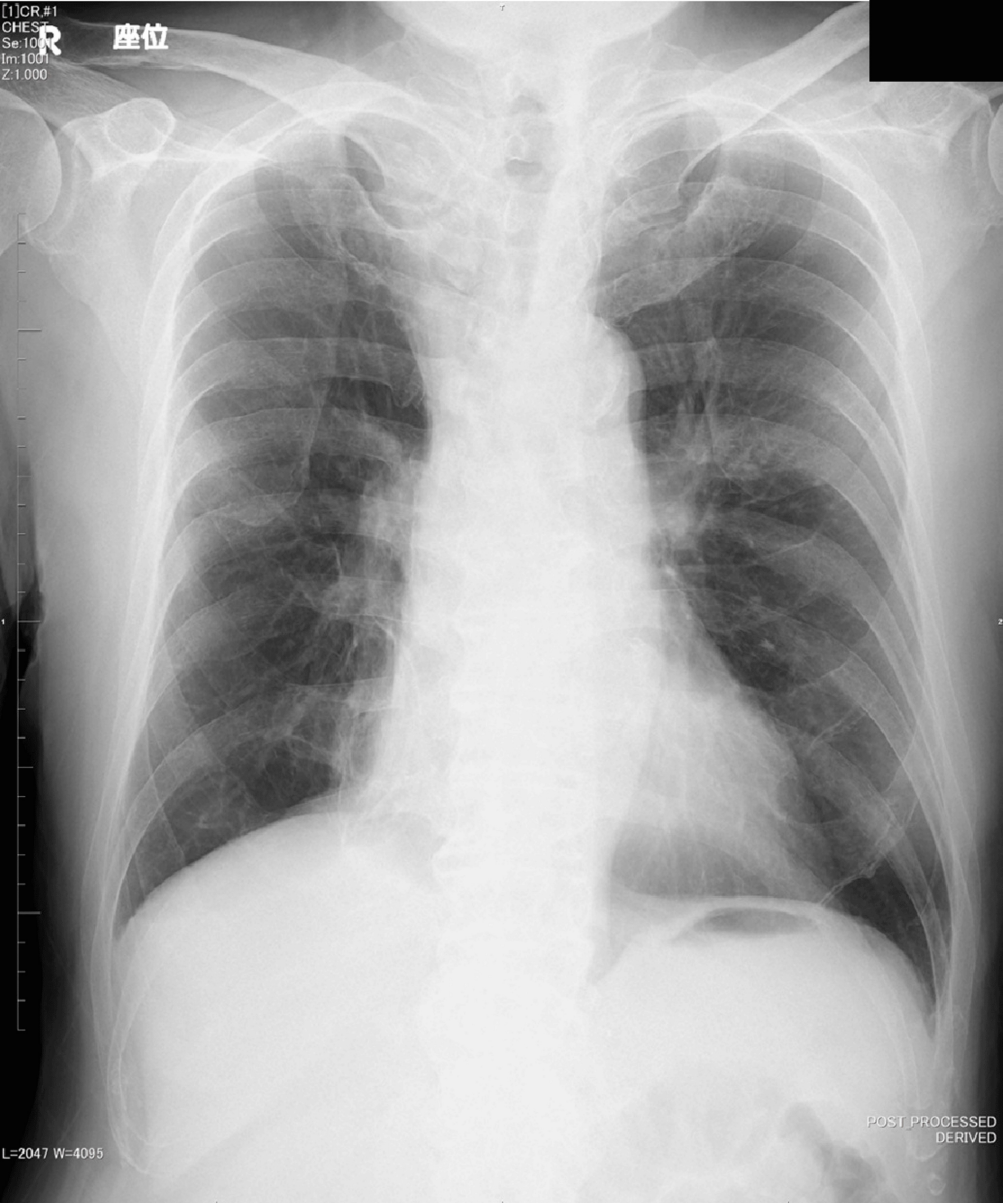
Chest X-ray on day 15. Chest X-ray showing with and without infiltration. No new areas of infiltration was noted.

On day 14, the patient experienced loss of appetite, and by day 15, he developed vertigo and vomiting with positional changes. A brain CT showed no evidence of intracranial disease, and a chest X-ray did not reveal any significant findings suggestive of pneumonia (Figure 2). On day 27, the patient developed a fever again, and auscultation revealed adventitious lung sounds, suggesting a recurrence of pneumonia. The ABPC/SBT regimen at 6 g/day was resumed, and the high fever subsided by day 30. Antibiotics were discontinued once again on day 33 after his condition improved, but his poor oral intake persisted. Trial home visits were conducted on days 36 and 37, as well as on days 51 and 52. During these periods, the patient was able to manage some daily activities and maintain oral intake to a certain extent. Based on this, transitioning to outpatient care following discharge was considered.

**Figure 3:**
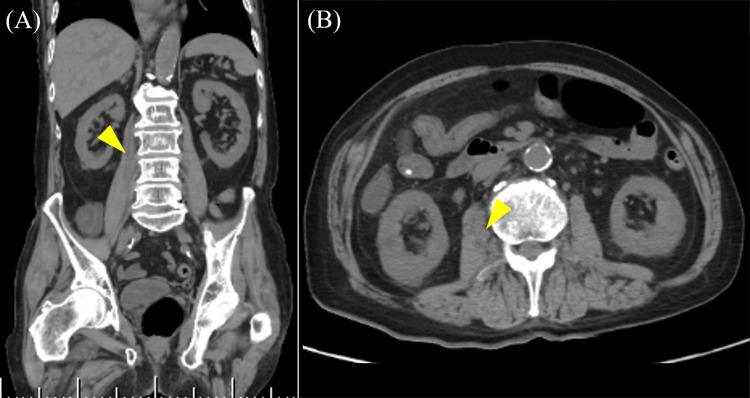
Abdominal CT imaging on day 59. Abdominal CT showing hypertrophy of the right iliopsoas muscle and a low-density area inside the right iliopsoas muscle. (A) Coronal section. (B) Axial section.

**Figure 4:**
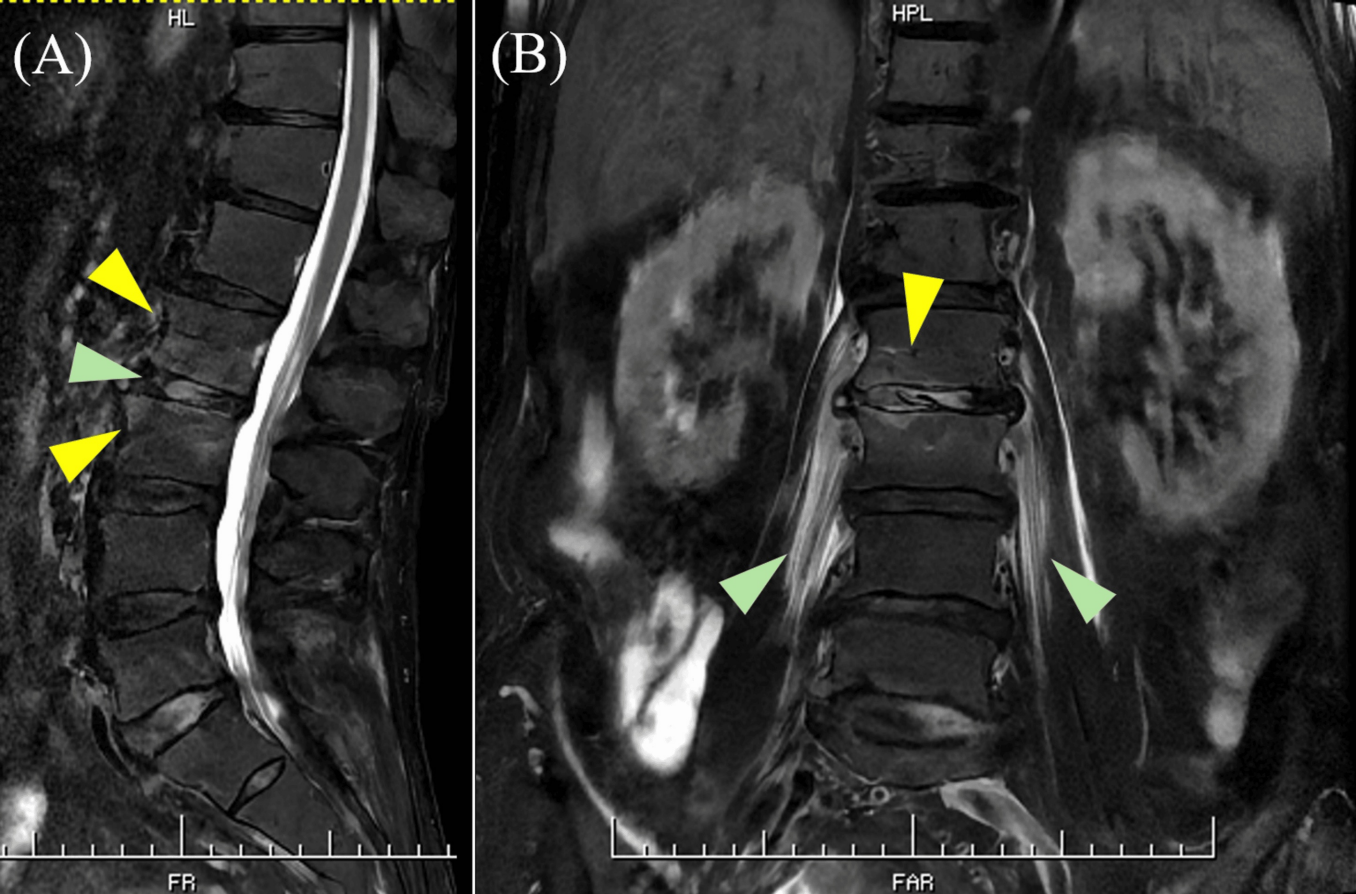
T2-weighted lumbar magnetic resonance imaging (MRI) on day 60. (A) Sagittal section of lumbar MRI on T2-weighted imaging showing hyperintensities at the L2 and L3 vertebral bodies (yellow arrowheads) and the L2/3 disc (green arrowhead). (B) Coronal section of lumbar MRI on T2-weighted imaging showing hyperintensities in the bilateral iliopsoas muscles (green arrowheads), the L2/3 disc (yellow arrowhead), and the L2 and L3 vertebral bodies (yellow arrowhead).

On day 57, the patient developed chills, fever, lower back pain, and bowel incontinence, prompting the restart of ABPC/SBT at 6 g/day. On day 59, blood tests revealed elevated lactate dehydrogenase (239 IU/L) and C-reactive protein (15.08 mg/dL), along with a decreased red blood cell count (374×10⁴/μL), hemoglobin (11.4 g/dL), and albumin (3.2 g/dL) (Table 2). Additionally, urinalysis revealed hematuria. On day 60, an abdominal CT scan showed a low-density area suggestive of an abscess in the right iliopsoas muscle (Figure 3). In addition, two sets of blood cultures taken on day 57 were positive for *P. aeruginosa*. In light of these findings, the antibiotic regimen was changed to meropenem (MEPM) at 3 g/day. On day 61, lumbar MRI showed hyperintensity at the L2/3 disc and the L2 and L3 vertebral bodies, suggestive of discitis and vertebral osteomyelitis (Figure 4). The antibiotic regimen was changed to ciprofloxacin (CPFX) at 800 mg/day on day 62. Despite ongoing treatment, his fever did not resolve. Percutaneous and surgical drainage were considered but were deemed unfeasible due to the size and location of the abscess. On day 82 of hospitalization, blood tests showed an elevated CRP level of 13.69 mg/dL, indicating persistently high inflammatory markers. The patient's body temperature fluctuated between 37.0-38.0°C without improvement. On day 86, we switched the antibiotic to cefepime (CFPM) at 2 g/day, but no significant change in the patient's fever was observed. On day 96, the patient exhibited increased sputum production, and blood tests showed a further rise in CRP to 21.69 mg/dL. A chest X-ray revealed infiltrates in the lower lung fields, leading to a diagnosis of worsening aspiration pneumonia. The antibiotic was then changed to piperacillin/tazobactam (PIPC/TAZ) at 13.5 g/day. Following this change, the patient's fever showed a slight improvement, ranging from 36.6-37.1°C, but their general condition, including poor appetite, remained unchanged. On day 102 of hospitalization, due to the complexity of his clinical condition and its deterioration, the patient was transferred to a chronic care facility for palliative management.

**Table 2:** The results of blood tests on day 59.

Inspection items	Result	Reference range
White blood cell count	8300 /μl	(3300-8600)
Red blood cell count	374×104 /μl	(435-555×104)
Hemoglobin	11.4 g/dl	(13.7-16.8)
Blood platelet	11.3×104 /μl	(15.8-34.8)
Total protein	7.2 g/dl	(6.6-8.1)
Albumin	3.2 g/dl	(4.1-5.1)
Alkaline phosphatase	80 U/l	(38-113)
Aspartate aminotransferase	21 U/l	(13-30)
Alanine aminotransferase	14 U/l	(10-30)
Lactate dehydrogenase	239 U/l	(124-222)
Creatine kinase	134 U/l	(59-248)
γ-glutamyltransferase	35 /U/l	(13-64)
Total bilirubin	1.3 mg/dl	(0.4-1.2)
Amylase	43 U/l	(44-132)
Blood urea nitrogen	9.9 mg/dl	(8.0-20.0)
Choline esterase	122 U/l	(240-486)
Creatinine	0.73 mg/dl	(0.65-1.07)
Sodium	137 mmol/l	(138-145)
Potassium	3.7 mmol/l	(3.6-4.8)
Chlorine	102 mmol/l	(101-108)
C-reactive protein	15.08 mg/dl	(0.00-0.14)

## Discussion

We describe a rare case of IPA related to *P. aeruginosa*, which was confirmed through CT imaging and blood cultures. In addition, MRI findings strongly suggested discitis at the L2/3 level and vertebral osteomyelitis at the L2 and L3 vertebral bodies. The patient repeatedly received ABPC/SBT for recurrent aspiration pneumonia before *P. aeruginosa* was isolated. Long-term exposure to antibacterial agents with no activity against *P. aeruginosa* may cause bacterial replacement, leading to *P. aeruginosa *infections. Based on his clinical course, since *P. aeruginosa* was isolated from the patient's blood while he was suffering from recurrent aspiration pneumonia, we speculate that *P. aeruginosa* entered the body through the lungs, leading to a bloodstream infection and caused IPA, discitis, and vertebral osteomyelitis. Alternatively, the complications of IPA, discitis, and vertebral osteomyelitis have been documented [13], and the primary focus may originate from any one of these three conditions and spread to nearby tissues. After confirming *P. aeruginosa *bacteremia, antimicrobial therapy was switched to antibiotics with anti-pseudomonal activity, including MEPM, CPFX, CFPM, and PIPC/TAZ. Due to the size and location of the abscess, drainage could not be performed. Despite these treatments, the patient did not improve and was transferred to another facility for palliative care.

To the best of our knowledge, there are only three previous reports of IPA related to *P. aeruginosa*. In two out of three papers, it has been reported that *P. aeruginosa* or *Pseudomonas* spp. were isolated in a summary of multiple IPA cases [5, 6]. However, no detailed clinical information on IPA related to* P. aeruginosa *was provided [5, 6]. In the remaining paper, Debes *et al. *described a case of a 72-year-old male with perforation of a sigmoid colon diverticulum, resulting in IPA caused by *P. aeruginosa* and Group B *Streptococcus *[12]. In this case, there were several risk factors for a compromised host, including advanced age, chronic obstructive pulmonary disease (COPD), diabetes mellitus, and mild chronic kidney disease. The patient exhibited few signs of fever or gastrointestinal symptoms but experienced left hip tenderness and restricted range of motion in the left hip. A CT scan revealed a left psoas abscess measuring approximately 9 × 6 × 18 cm. Antimicrobial therapies with ABPC/SBT and levofloxacin (LVFX), along with CT-guided drainage, were administered. The culture of the purulent material identified Group B *Streptococcus* and *P. aeruginosa*. Subsequently, an exploratory laparotomy and diverting ileostomy were performed. His prognosis was not described in the report.

In comparison with the previously reported case, both cases were elderly and compromised hosts. In the previous case, perforation of the diverticulum caused inflammation and bacteria spread to the adjacent iliopsoas muscle, leading to IPA. In our case, however, the primary focus remains unknown. Hematogenous spread from aspiration pneumonia to the iliopsoas muscle could have resulted in IPA, or the primary focus may have been IPA, discitis, or vertebral osteomyelitis, with subsequent spread to neighboring tissues. The causative pathogens in the reported case were *P. aeruginosa* and Group B *Streptococcus*, which were isolated from purulent discharge via drainage, whereas in our case, *P. aeruginosa *was isolated from a blood culture. Regarding treatment, the antibiotics used in the reported case were ABPC/SBT and LVFX, while in our case, we used ABPC/SBT, MEPM, CPFX, CFPM, and PIPC/TAZ. While drainage and surgical intervention were performed in the previous report, no surgical interventions were performed in our case, which may have contributed to the poor prognosis. Although there are few reports of IPA caused by *P. aeruginosa*, both cases highlight that *P. aeruginosa* can be a causative pathogen of IPA.

Our patient had a poor prognosis, and we discussed potential reasons for this outcome. The patient had several risk factors for infection, including malnutrition, type 2 diabetes, and advanced age, all of which likely contributed to increased susceptibility to infection. It is generally recognized that infections caused by *P. aeruginosa* carry a higher risk of severe outcomes compared to other pathogens [11]. Given these factors, it is plausible that the unfavorable clinical course observed in this case might be influenced by the patient’s underlying conditions. Malnutrition, as an intervenable factor, was present; however, the patient refused proactive nutritional interventions, including enteral nutrition via a gastrostomy or nasogastric tube, as well as total parenteral nutrition, necessitating the continuation of peripheral parenteral nutrition. Moreover, our case involved a prolonged hospital stay and extended use of broad-spectrum antibiotics, which led to bacterial replacement by drug-resistant organisms such as *P. aeruginosa*.

However, we did not switch to antibiotics with anti-pseudomonal activity until *P. aeruginosa* was detected by blood cultures, which may have contributed to the development of IPA related to *P. aeruginosa* and the unfavorable outcome in this case. In addition, drainage was not performed in our case. This procedure may have contributed to not only the treatment but also the detection of causative pathogens, including occasionally mixed pathogens [4]. From the perspective of switching to targeted therapy, drainage of the abscess should be considered whenever possible to facilitate the identification of the responsible pathogens. It is possible that inadequate source control due to the lack of drainage, and inadequate targeted treatment due to the potential presence of other causative bacteria besides *P. aeruginosa*, may have contributed to the poor prognosis. Alternatively, the effectiveness of drainage and surgical interventions for IPA remains unclear. In a study involving 120 cases of IPA, 29 out of 36 patients (80.6%) treated with antibiotics alone achieved a cure, with four patients (11.1%) experiencing relapse and 3 patients (8.3%) dying [5].

In the group treated with both antibiotics and percutaneous drainage, 49 out of 63 patients (77.8%) were cured, 11 patients (17.4%) relapsed, and three patients (4.8%) died. Meanwhile, in the group receiving antibiotics combined with surgical intervention, 17 out of 21 patients (81%) were cured, four patients (19%) relapsed, and no deaths were reported [5]. These results suggest that none of the treatment modalities significantly influenced mortality [5]. Additionally, there were no substantial differences in relapse rates between the groups [5]. A simple comparison might indicate that the antibiotics-alone group had a slightly better success rate and lower relapse rate compared to the drainage group. However, these findings are likely subject to bias. It is plausible that the antibiotics-only group included a higher proportion of cases with smaller abscesses where drainage was unnecessary or unfeasible, while the drainage group might have included more severe cases. Therefore, no definitive conclusion has been reached regarding the efficacy of antibiotics alone versus a combination with drainage. In our case, due to the small size of the abscess and the technical difficulty of performing drainage, as well as the lack of clear evidence supporting the efficacy of drainage, we chose not to perform the procedure and opted for treatment with antibiotics alone.

## Conclusions

We present a rare case of IPA, discitis, and vertebral osteomyelitis related to *P. aeruginosa*. In cases involving the combination of* P. aeruginosa* and IPA, various compromised host factors and *P. aeruginosa* itself might contribute to the adverse outcome. This report may enhance our understanding of the relationship between IPA and *P. aeruginosa* infections. The key lesson from our case is that *P. aeruginosa* can be a causative pathogen of psoas abscesses. Elderly patients with prolonged hospitalization and a history of extended exposure to antibiotics are at significant risk for *P. aeruginosa* infections. For such patients, the use of antibiotics with antipseudomonal activity should be considered. Further studies and reports are needed to determine more effective treatments and better prognostic outcomes for *P. aeruginosa*-related IPA.
